# Membranous nephropathy associated with type 1 autoimmune pancreatitis and dominant glomerular IgG4 deposit

**DOI:** 10.1007/s13730-013-0077-y

**Published:** 2013-05-03

**Authors:** Shinichi Sueta, Makiko Kondo, Takeshi Matsubara, Yumiko Yasuhara, Shinichi Akiyama, Enyu Imai, Hisashi Amaike, Miho Tagawa

**Affiliations:** 1 Department of Nephrology, Kyoto Katsura Hospital, 17 Yamada-Hirao-cho, Nishikyo-ku, Kyoto, 6158256 Japan; 2 Department of Nephrology, Kyoto University Graduate School of Medicine, 54 Kawahara-cho, Shogoin, Sakyo-ku, Kyoto, 6068507 Japan; 3 Department of Diagnostic Pathology, Kyoto Katsura Hospital, 17 Yamada-Hirao-cho, Nishikyo-ku, Kyoto, 6158256 Japan; 4 Department of Nephrology, Nagoya University Graduate School of Medicine, 65 Tsurumai, Showa-ku, Nagoya, Aichi 4668550 Japan; 5 Department of Surgery, Kameoka Municipal Hospital, 1-1 Shino-Noda, Shino-cho, Kameoka, Kyoto 6218585 Japan

**Keywords:** Membranous nephropathy, IgG4-related disease, IgG4

## Abstract

We report a case of membranous nephropathy associated with type 1 autoimmune pancreatitis. A 58-year-old man presented with anorexia. Work-up revealed a mass in the pancreatic head, which was subsequently resected. Pathological examination showed diffuse infiltration of immunoglobulin (Ig) G4-positive plasma cells, which was compatible with the diagnosis of type 1 autoimmune pancreatitis. Serum IgG4 was elevated. He developed nephrotic syndrome around the time of the surgery. Kidney biopsy confirmed the diagnosis of membranous nephropathy. Immunofluorescent staining showed predominant glomerular IgG4 deposit among IgG subclasses. Tubulointerstitial nephritis, which is usually a dominant feature of renal involvement in IgG4-related disease, was not observed. The patient was treated with prednisolone and several immunosuppressants. During the course, the degree of proteinuria was associated with the serum IgG4 level. Serum antibody against phospholipase A2 receptor was negative. These findings together with IgG4-dominant glomerular deposit suggest that IgG4 may play a unique role in the pathogenesis of secondary membranous nephropathy caused by IgG4-related diseases.

## Introduction

Immunoglobulin (Ig) G4-related disease (IgG4-RD) is characterized by increased serum IgG4 levels, caused by the infiltration of IgG4-positive cells into various organs [1–3]. Renal involvement has been reported in 9–15 % of IgG4-RD [4, 5]. In the latest review [6], 37 cases of IgG4-related kidney disease were reviewed. In all cases, tubulointerstitial nephritis (TIN) was a dominant feature and glomerular involvement was reported in 24 %: 3 cases with membranous nephropathy (MN), 1 with membranoproliferative glomerulonephritis, 4 with mesangial proliferative glomerulonephritis, and 1 with endocapillary proliferative glomerulonephritis. Although IgG4-related kidney disease does not include cases in which the glomerular lesion is the sole kidney lesion without TIN [7, 8], a literature review revealed two case reports in which glomerular lesions were the only renal abnormalities associated with IgAG4-RD [9, 10]. In these case reports, MN was a dominant feature [9, 10]. MN associated with IgG4-RD has been attracting attention for several reasons. IgG4 deposits predominantly among IgG subclasses in idiopathic MN (IMN) [11]. M-type phospholipase A2 receptor (PLA2R) was identified as a possible target antigen in IMN and autoantibodies against PLA2R detected in serum samples from patients with IMN were mainly of IgG4 subclass [12]. Furthermore, type 2 helper T cells produce cytokines which stimulate B cells to produce IgG4 in both IgG4-RD [13] and IMN [14]. On the other hand, the serum IgG4 level is not elevated in IMN [11]. Also, antibodies against PLA2R were not detected in IgG4-RD [15].

We describe a unique case of MN associated with IgG4-RD. The patient presented with IgG4-dominant deposit in the glomerular capillary wall and no associated TIN. The degree of proteinuria and serum IgG4 levels were associated and serum anti-PLA2R antibody was negative.

## Case report

**Fig. 1:**
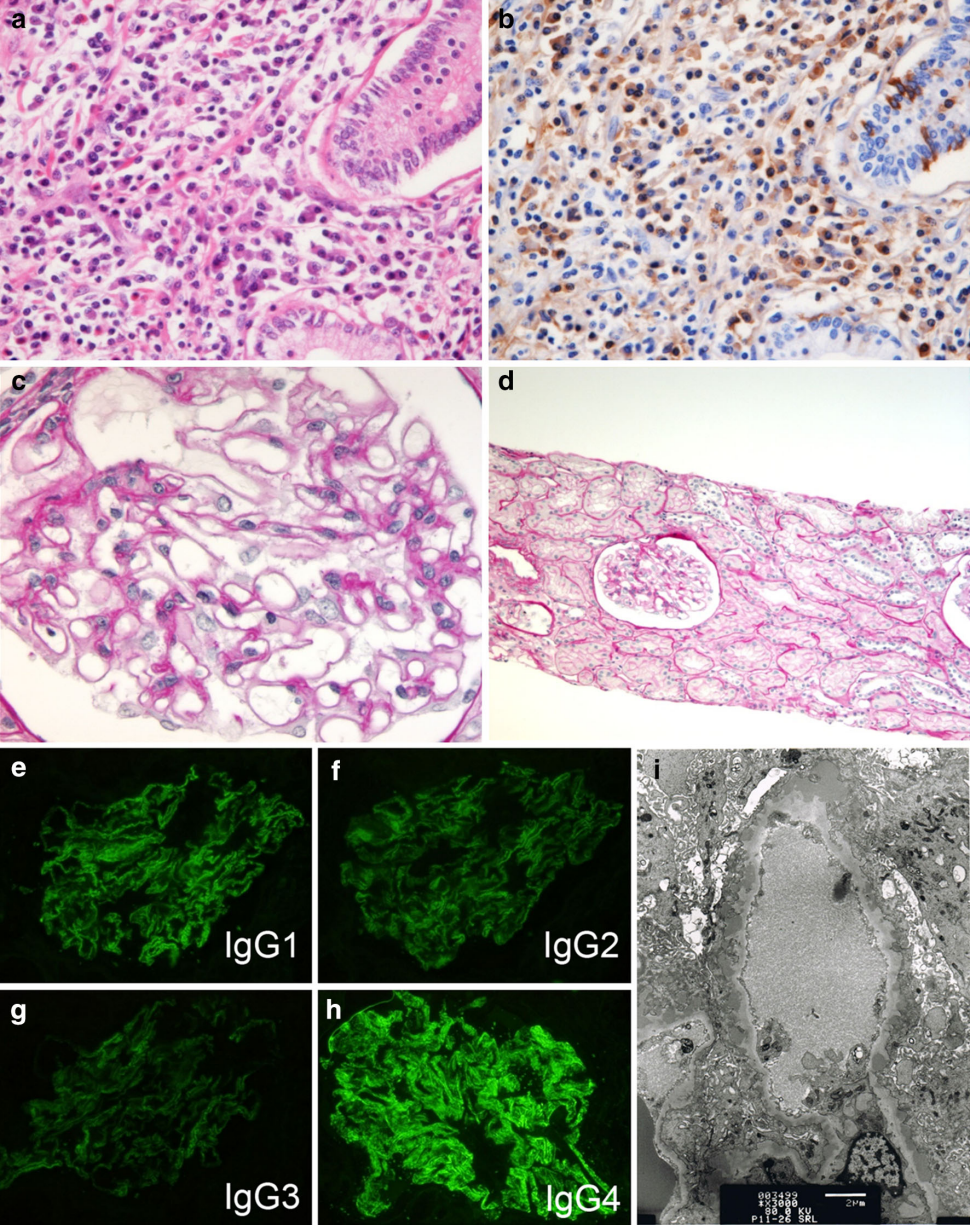
**a**, **b** Pancreatic tissues, **c**–**i** kidney biopsy. **a** Diffuse infiltration of plasma cells and lymphocytes in pancreatic tissue (H&E, ×40). **b** Immunohistochemistry on paraffin tissue for IgG4: the stain showed diffuse and dense labeling of infiltrated plasma cells (×40). **c** Well-preserved structure of glomeruli were observed by light microscopic examination of the renal biopsy (periodic acid-Schiff stain, ×40). **d** No cellular infiltration was seen in the interstitium (periodic acid-Schiff stain, ×10). **e**–**h** Immunofluorescence staining showed IgG4-dominant deposition in the glomerular basement membrane. **i** Electron microscopy showed subepithelial deposits

The patient is a 58-year-old Japanese man with no previous medical history. In September 2010, he presented with anorexia. He was not on any medications. Serum transaminase and alkaline phosphatase levels were elevated. Urinalysis, renal function, serum total protein, and albumin levels were as follows: urinalysis: 3+ protein and 3+ occult blood, blood urea nitrogen 17 mg/dL (6.0 mmol/L), serum creatinine 0.67 mg/dL (51.1 μmol/L), total protein 6.2 g/dL (62 g/L), albumin 3.1 g/dL (31 g/L). Enhanced computed tomography of the abdomen showed localized tumor on the pancreatic head with no kidney abnormalities. Magnetic resonance cholangiopancreatography showed tumor on the pancreatic head without pancreatic duct dilation and stenosis. He underwent subtotal stomach-preserving pancreatoduodenectomy for localized tumor on the pancreatic head. Histological examination of the pancreas showed diffuse infiltration of plasma cells and lymphocytes without evidence of malignancy (Fig. 1a, b). There were characteristic storiform fibrosis and obliterative phlebitis. Immunohistochemistry for IgG4 showed more than 10 labeling plasma cells in the high power field (Fig. 1a, b). The histological characteristics met the diagnostic criteria for type 1 autoimmune pancreatitis by the Ministry of Labor, Health and Welfare of Japan [16].

After the surgery, the patient developed ascites and lower extremity edema. His serum albumin level was 1.7 g/dL (17 g/L) and 24-h urinary protein excretion was 15.7 g. He also developed portal vein thrombosis.

In December, he was transferred to our hospital for the evaluation of nephrotic syndrome. On admission, he was taking candesartan, furosemide, spironolactone, warfarin, aspirin, famotidine, and voglibose. On examination, he was normotensive and had bilateral lower extremity edema. Urinalysis showed 4+ proteinuria and 2+ occult blood. Other laboratory data were as follows (reference range in parentheses): blood urea nitrogen: 13 mg/dL (4.6 mmol/L), serum creatinine: 0.7 mg/dL (53.4 μmol/L), albumin: 1.8 g/dL (18 g/L), total cholesterol: 140 mg/dL, C-reactive protein: 0.01 mg/dL (9.52 nmol/L), hepatitis B surface antigen: negative, hepatitis C antibody: negative, antinuclear antibody: negative, C3: 85.3 (65–135) mg/dL, C4: 22.0 (13–35) mg/dL, IgG: 934 (870–1700) mg/dL (9.34 g/L), IgA: 148 (110–410) mg/dL (1480 mg/L), and IgM: 70 (35–220) mg/dL (700 mg/L). His serum IgG4 level was elevated to 377 (8–105) mg/dL.

In January 2011, a renal biopsy was performed. Light microscopy showed normocellular glomeruli with mild thickening of the glomerular capillary walls and normal mesangial matrix (Fig. 1c). There was no cellular infiltration in the tubulointerstitium (Fig. 1d). Immunofluorescence staining showed deposition of IgG and C3 in the glomerular basement membrane. Intense IgG4 staining was observed along the glomerular capillary walls and was absent in other components (Fig. 1e–h). Staining for IgG1, IgG2, and IgG3 was weak (Fig. 1e–h). Staining for IgA and IgM was negative. Tubular basement membrane staining was negative for all the tested immunoglobulins. Electron microscopy showed subepithelial electron-dense deposit (Fig. 1i). Histological features were compatible with the diagnosis of MN.

Enzyme-linked immunosorbent assay for PLA2R was performed using 0.5 μg of recombinant human PLA2R (21–663 amino acid) per well as an antigen. The patient’s plasma diluted 1:1000 was incubated with the antigen. After washout, horseradish peroxidase-conjugated anti-human IgG1–G4 diluted 1:1500 was added (IgG1, 3, 4: Abcam, Tokyo, Japan and IgG2: Invitrogen, Yokohama, Japan). Anti-PLA2R antibodies were not detected in the patient’s serum. Absorbance at 450 nm was 0.093 for IgG4 [negative control (healthy person) 0.050, positive control (a patient with IMN) 1.677]. Absorbance at 450 nm for IgG1–3 was not significantly elevated compared with the negative control.

**Fig. 2:**
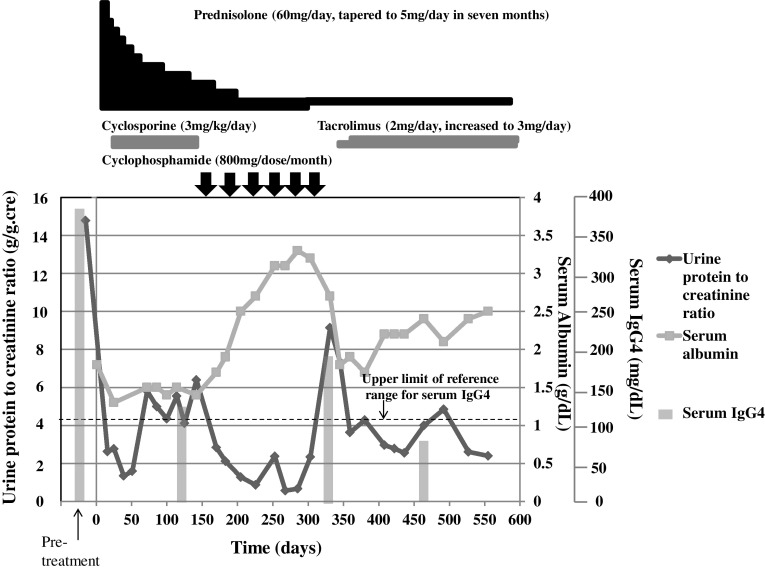
The clinical course of membranous nephropathy (MN) and change in serum IgG4. The serum IgG4 decreased in association with the resolution of proteinuria

The patient was treated with prednisolone (60 mg/day, tapered to 5 mg/day in 7 months) and cyclosporine. There was improvement in the urine protein to creatinine ratio, but serum albumin remained low (Fig. 2). Three months later, cyclosporine was discontinued and monthly intravenous pulse cyclophosphamide was started (Fig. 2). After the initiation of cyclophosphamide, there was improvement in the urine protein to creatinine ratio and serum albumin. His serum IgG4 level decreased to 117 mg/dL, concomitantly with the improvement in proteinuria (Fig. 2). Eleven months after presentation, he experienced recurrence of nephrotic syndrome. His serum IgG4 level increased to 187 mg/dL, concomitantly with the increase in proteinuria (Fig. 2). After the recurrence, the patient was treated with prednisolone and tacrolimus. There was improvement in the urine protein to creatinine ratio and his serum IgG4 level was 75.6 mg/dL (Fig. 2).

## Discussion

We reported a case of MN associated with IgG4-RD. This case has several distinct features compared with previous reports: (1) the severity of proteinuria was associated with the serum IgG4 level; (2) anti-PLA2R antibody was negative; (3) immunofluorescence staining showed IgG4-dominant deposition among IgG subclasses in the glomerular capillary wall; (4) no associated TIN was observed.

A literature review revealed 10 cases of biopsy-proven MN associated with IgG4-RD [9, 10, 17–24]. A clinical feature of MN associated with IgG4-RD is that the amount of proteinuria is variable (24-h urine protein excretion ranging from 1.4 to 15 g/day [9, 10, 17–24]), and that it is associated with variable extra-renal involvement (5 with pancreatitis [9, 19–21, 24], 1 with possible pulmonary involvement [21], 1 with prostatitis [24], 1 with lymph node involvement [24], and 1 with periaortitis [10]), and that about half will respond well to steroid mono-therapy and the rest seems to be refractory [9, 10, 17–24]. In our case, the patient presented with type 1 autoimmune pancreatitis and MN. Twenty-four-hour proteinuria was 15.7 g. The patient achieved partial remission after multiple combinations of immunosuppressants and steroids.

A pathological feature of renal involvement of IgG4-RD is usually TIN, and MN was observed in only 8 % of cases [6]. As far as we know, in the literature concerning MN associated with IgG4-RD, only 2 cases were without TIN [9, 10], and in one of them, IgG4 deposit was seen in the tubular basement membrane, suggesting some tubular involvement [10]. In 7 cases examined with immunofluorescence staining for IgG subclasses, IgG4 staining was dominant in glomeruli in 4 [10, 21–23], IgG2 and IgG3 were dominant in 2 cases [9, 18], and no glomerular staining was seen in one case [19]. IgG4 staining was also observed in plasma cells in the interstitium [17, 21–23] or tubular basement membrane [10], except for one case [9]. Our case was not associated with TIN and IgG4-dominant staining was seen only in the glomeruli, not in the interstitium. During renal biopsy, three cores of specimens were obtained, most of which were cortex, and TIN was not observed in any of the specimens. Also, enhanced computed tomography did not show focal regions in the kidneys. The possibility of sampling error is unlikely.

MN could be idiopathic or secondary to various diseases. The diagnosis of IMN is usually by the exclusion of secondary causes, but several features could help the differentiation of idiopathic from secondary MN. Anti-PLA2R antibody was positive in 68.5–81.7 % of IMN, whereas it was positive in only 0–12.2 % of secondary MN [12, 26–28]. However, in these studies, the possibility could not be excluded that the coincidental occurrence of IMN with the associated disease was considered to be secondary [25–27]. Immunofluorescence study in IMN usually shows IgG4-dominant glomerular deposit, whereas IgG2 or IgG3 is dominant in secondary MN [28]. The absence of circulating anti-PLA2R antibody was suggestive of secondary MN in our case. Also, the association of serum IgG4 levels and the degree of proteinuria in our case suggests secondary MN. The level of serum IgG usually decreases in a background of nephrotic syndrome, but in our case, the IgG4 levels increased at the onset and during the relapse of nephrotic syndrome. This even more strongly suggests the association of serum IgG4 levels and the degree of proteinuria due to membranous nephropathy, although more cases need to be studied in order to draw definite conclusions. Interestingly, immunofluorescence microscopy showed intense IgG4 staining along the glomerular capillary walls in our case, which is usually a feature of IMN. Cravedi et al. [9] also reported a case in which increased serum IgG4 was followed by the onset of MN and anti-PLA2R was negative. In their report, the serum IgG4 level decreased after the treatment with rituximab, but proteinuria persisted and IgG3 staining was dominant in the glomeruli, observations which are different from our case.

In summary, we reported a case of MN without TIN associated with IgG4-RD. The association of serum IgG4 and proteinuria and negative anti-PLA2R antibodies suggested that MN was secondary to IgG4-RD. There was dominant IgG4 deposit in the glomeruli. Our case suggests the possible role of IgG4 in the pathogenesis of MN more clearly than previous cases. The antigen against IgG4 in our case needs to be identified in a future study.
